# Problems and countermeasures associated with intercultural adaptation in international education according to the communication action theory model

**DOI:** 10.3389/fpsyg.2022.942914

**Published:** 2022-08-29

**Authors:** Yanjin Liu, Yun Song, Yun Yan

**Affiliations:** ^1^College of Educational Sciences, Xinjiang Normal University, Urumqi, China; ^2^Sichuan International Education Development Research Center, Chengdu, China; ^3^School of Preparatory Education, Aba Teachers University, Shuimo, China; ^4^College of Marxism, Xihua University, Chengdu, China

**Keywords:** communication action theory, international education, intercultural adaptation, psychological adaptation, intensification of international capital

## Abstract

Due to the development of the Chinese economy, the consolidation of national power worldwide, and the increasing frequency of economic and cultural exchanges with foreign countries, the number of people from various countries who travel to China to engage in exchanges has increased significantly. Given the development of economic globalization, the acceleration of the process of educational internationalization represents a general trend in higher education development and a common requirement for universities. In addition to education, international students also experience cross-cultural adaptation, that is, behavioral adaptation or changes that occur in people in response to changes in their home country, whether in the form of external or internal cultural adaptation. For international students, the problem of cross-cultural adaptation not only hinders their learning progress but also affects their psychology and living conditions. This article explains the construction of Habermas’s theory of communication action. According to this theory, the purpose of communication is to coordinate the common actions of actors, and this coordination is achieved through mutual communication, which is mediated by language. The article also discusses the cross-cultural adaptation experienced by international students in Chinese universities and highlights the importance of developing educational services for international students in the context of international education. Studies have shown that female students exhibit slightly worse psychological adaptation than male students and female students have a slightly higher rate of depression rate than male students. An interpersonal study of 120 international students traveling to China found that most of these students (60%) were able to adapt in terms of their interpersonal relationships. To solve the problems associated with cross-cultural adaptation of international students in China, some countermeasures have been proposed, mainly including active participation in interpersonal communication, an enhanced understanding of the new culture, and the amplification of cultural identity.

## Introduction

Due to the progress of China’s reform and opening-up and the intensification of the flow of international capital and talent, primary and secondary education in China has become increasingly internationalized and globalized. Since the beginning of the 21st century, an increasing number of foreign students and students from Hong Kong, Macao, and Taiwan have attended primary and secondary schools in mainland China for various reasons, such as because their parents are engaged in business on mainland China, because their families have relocated there, or because they aspire to and identify with mainland China and its approach to education. Their cultural adaptability has become an important aspect of school management.

In addition to the individual characteristics of these students, the values, ways of thinking, and behavioral concepts that are formed by the long-term influence of their parent cultures are also very different. After studying abroad, changes in the surrounding environment represent a new challenge for international students to adapt to social activities and campus learning in the context of Chinese culture. If cross-cultural adaptation cannot be achieved, problems in students’ study and life can result, such as autism, low mood, or irritability. In severe cases, this situation may lead to depression, suicide, and other tragedies. In the context of learning, other results may include inattention in class, not adapting to the teacher’s teaching method, failing to pass exams, or even dropping out of school. The problem of cross-cultural adaptation not only affects the success or failure of the international student’s own study but also affects the international student’s evaluation of the host country after returning home, thus affecting China’s image in the international arena. Of course, the problems caused by cultural maladjustment have inevitably hindered the continuous development of education in China. Based on the practical work conducted in this context, this study tries to delve more deeply into the student group to understand the problems associated with the cross-cultural adaptation of international students in China and to find reasonable suggestions for improving the cross-cultural adaptation of international students in China.

The article took international students from China as its research object and drew on relevant theories of cross-cultural adaptation. Questionnaire analysis revealed that in the analysis of the adaptation of international students to the living environment on campus, in terms of the three indicators of canteen environment, lunch quality, and dining order, a large number of students were relatively satisfied with their dietary conditions, and only 24.1% of the total (29 people) was very dissatisfied with their lunches. Most of the students’ satisfaction with on-campus drinking water was also at an average level, and the students’ adaptability to on-campus medical treatment was unsuitable. In the analysis of the adaptation of international students to the education and teaching environment, in terms of teaching methods, 36.7% of the students were satisfied or very satisfied. In terms of course quality, 23.3% of the students considered themselves to be dissatisfied or very dissatisfied. This finding shows that students are unaccustomed to this aspect of education and teaching. In the analysis of the psychological characteristics of international students and their adaptation in terms of interpersonal communication, the psychological adaptation of girls was slightly worse than that of boys, and the levels of depression exhibited by girls were slightly higher than those exhibited by boys. Most international students (60%) were able to adapt to interpersonal relationships.

This paper is divided into six chapters. The first chapter is the introduction, which mainly summarizes the research background and significance of this paper.

The second chapter discusses related work and summarizes research results pertaining to the communication action theory model and cross-cultural adaptation in recent years.

The third chapter analyzes the definition of the communication action theory model and the process of cross-cultural adaptation in detail and conducts a principal component analysis to investigate the factors influencing foreign students’ cross-cultural adaptation.

The fourth chapter mainly develops the communication action theory model and designs a questionnaire survey to investigate students’ intercultural adaptation in the context of international education.

The fifth chapter mainly summarizes the cross-cultural adaptation of international education students and proposes countermeasures in accordance with the survey results.

The sixth chapter is the conclusion, which summarizes the main research work of this paper.

## Related work

Language plays an important role in communication action theory, and Jovanoski presented Jürgen Habermas’s epistemological perspective on deliberative democracy theory. Beginning in the 1970s, Habermas constructed a series of hypotheses concerning his communication action theory to overcome the crisis of legitimacy. This position stems from a critique of the deep gulf between the “constitutional-democratic legal order” as a normative framework and the ways in which forms of social power can be imposed to undermine legitimate law-passing processes. Habermas’s theories and arguments concerning communicative action and communication-based (democratic) political decision-making processes raised the possibility of breaking with procedural arguments for the representation of so-called overarching patterns of democratic practice. Habermas respected the difference between facts and norms in every contemporary legal concept (Jovanoski and Sharlamanov, 2021). Carvalho reflected on some of the assumptions and development of critical thinking associated with communication action theory in the context of training undergraduate nursing students. He proposed one possibility of developing critical thinking among students in these courses. Communication is thus understood as a continuous, dynamic, conversational process that is inherent in nurse training, in which context interventions are contextual and meaningful to students and thus contribute to promoting critical thinking (Paula et al., 2017). Jürgen Habermas’s two-volume theory of communication action simultaneously attempts to develop a socially based theory of action to replace the subjectivist and individualistic foundations of most social theories, i.e., one that is based on a “two-level social concept linking the ‘lifeworld’ and ‘system’ paradigms,” a critical theory of modernity that retains the enlightenment ideal of a society based on reason, and a theory of meaning that is rooted in the developmental logic of world-historical rationality. Habermas tried to find a moderate position between totalitarian closure and relativism, indicating why modernism’s project of universal rationality remains viable. Although Habermas’s work has received a great deal of attention, Halton E in particular is sympathetic to the questions raised by Habermas. However, there is evidence to suggest that his project is fundamentally flawed, since it uncritically assumes that only critical reason can provide a legitimate criterion for communicative reason. Rationality is far more expansive than this limited view. These flaws become particularly apparent when examining his analysis of myth, action, and lifeworld (Halton, 2019). Mckeever applied communication theory to explore and help explain public support for causes and organizations in the form of prosocial behaviors, including donations, volunteering, and participation in advocacy efforts. Mckeever’s study applied the situation theory of problem solving (STOPS) and found that the STOPS model was supported with respect to predicting communication actions. Situational positivity affects other behaviors, including positive forms of communication, financial support, volunteer support, and other forms of advocacy. The implications for practitioners managing communications or organizations involved in such work were discussed. That study provides theoretical value that contributes to the generalization of the STOPS model to communication scholars and discusses its practical implications for nonprofit organizations and other types of organizations, although its scope of application is not large (Mckeever et al., 2019). Duckett critiqued the transparency and legitimacy of participatory scenario planning based on Habermas’ “communication action theory,” considering a case study focused on the development of scenarios in the livestock sector in Scotland. Consider the extent to which this case study approximates the conditions of an “ideal discourse situation” and the ways in which these conditions can be applied more broadly to participatory scenario planning. The rationale for participatory scenario planning at the science-policy interface was explored, with strict reference to the corporate context in which scenario planning develops. The aim was to optimize its potential for application in the context of sociotechnical and environmental governance. The researchers’ collective reflections on the case studies were mapped onto an exponential matrix representing ideal speech conditions. A further category of analysis emphasized the degree of approximation of ideal speech. Although many of the constraints associated with achieving an ideal speech situation reflect the stubborn, practical logistics involved in organizing participatory exercises (Duckett et al., 2017). Although the theory of communication action has received a great deal of attention, it has not been studied in depth.

In China, research concerning intercultural adaptation has also been conducted in recent years. Despite the purposeful choice to travel abroad, international students continue to face significant intercultural conflicts in this context. The extant research pertaining to student diversity has not adequately taken into account international students, whose narratives remain silent in the context of diversity research. These complex intercultural adaptation processes were the subject of research for Resch, who addressed the most pressing areas of concern for international students. Participants were involved in the preliminary coding of the material, while the researchers continued to focus on coding. The findings showed that international students and staff experience problems in six areas: (1) differences in communication, (2) concerns regarding belonging and identity, (3) differences in hierarchies, (4) differences in cultural and gender roles, (5) differences in perceptions of time, and (6) the impact of colonialism. The research shed light on the narratives of international students and staff in these areas of concern, called for greater awareness and action, and suggested implications for practice. In particular, services are provided to students both before and after their travels to reflect on and assess key experiences for future study (Resch et al., 2021). Due to the growing popularity of studying abroad, the cross-cultural adaptation of students studying abroad has also become more important. Therefore, ways of improving the cross-cultural adaptation experience of international students are worth exploring. Focusing on the pretravel period, Fang investigated the ways in which Chinese higher education students prepare for cross-cultural learning prior to traveling abroad, the effects of their previous cross-cultural learning experiences on their cross-cultural adaptation and the manners in which their perceptions of those previous cross-cultural learning affected their ongoing experiences. By offering relevant recommendations for promoting prior cross-cultural learning, this research provided insights into the ways in which Chinese university students and other stakeholders who are involved in these students’ experiences studying abroad can use the time prior to traveling abroad to produce better outcomes in terms of overseas students’ cross-cultural adaptation (Fang et al., 2020). These studies have provided detailed analyses of communication action theory and the issues associated with intercultural adaptation. Undeniably, these studies have greatly promoted the development of the corresponding fields. People can learn a great deal from the methodology and data analysis used in these studies. However, relatively few studies have investigated intercultural adaptation from the perspective of communication action theory, and it is necessary to apply these theories to research in this field more fully.

## Intercultural adaptation in the context of international education according to the communication action theory model

### Theoretical model of communication action

Habermas’s theory of communication action is mainly used to solve a dilemma in the context of contemporary social processes: the contradiction between formal rationality and substantive rationality. Understanding and exploring Habermas’s theory of communication action from a new perspective can not only broaden our horizons but also contribute to the construction of our proposed harmonious society (Arnold et al., 2020).

Communicative actions are actions that involve communication among people through the use of language. The purpose of communication is to allow actors to coordinate their actions with each other, and this collaboration is achieved through mutual communication using language as a medium of communication. Action subjects can interact in an ideal speech situation in the life world *via* language and discuss each other’s goals, means, and forms in the context of this interaction. Communication action is mediated by language, so it should be analyzed from the perspective of language (Shin, 2021). The smooth progress of communication action depends on the existence of a free and equal communication environment. Such an environment is determined by whether the communicator has the will to communicate sincerely, that is, whether the participants in the communication action have the same discursive power, participate in the argumentation of discourse freely and equally, and do not care about their respective identities and occupations. Each subject no longer has various halos, and even vulnerable groups can reasonably protect their rights. On the basis of the achievements of his predecessors in linguistic analysis, Habermas proposed his own universal pragmatics. The task of universal pragmatics is to indicate the conditions under which speech acts can achieve their purpose, the basis of discourse validity, and the conditions under which communicative acts can be performed successfully (Awan et al., 2020).

The purpose of Habermas’s theory of communication action is to establish a universal criterion that can be used to describe, analyze, criticize and evaluate modern society. This theory shows that the contradiction between the form and the content of modern society can be resolved and proposes communication action as a solution (Buhmann et al., 2019). Habermas’s concept of communication action reveals one of the most fundamental aspects of human action, that is, mutual understanding through verbal communication. In addition, humans coordinate their actions in accordance with this understanding, and behavior related to such mutual understanding among subjects serves as the basis of this theory. With respect to the cross-cultural adaptation of international students who travel to China, the theory of communication action provides a regular context for interpersonal dialog and mutual understanding across different cultures (White, 2022).

### Process of cross-cultural adaptation

**Figure 1:**
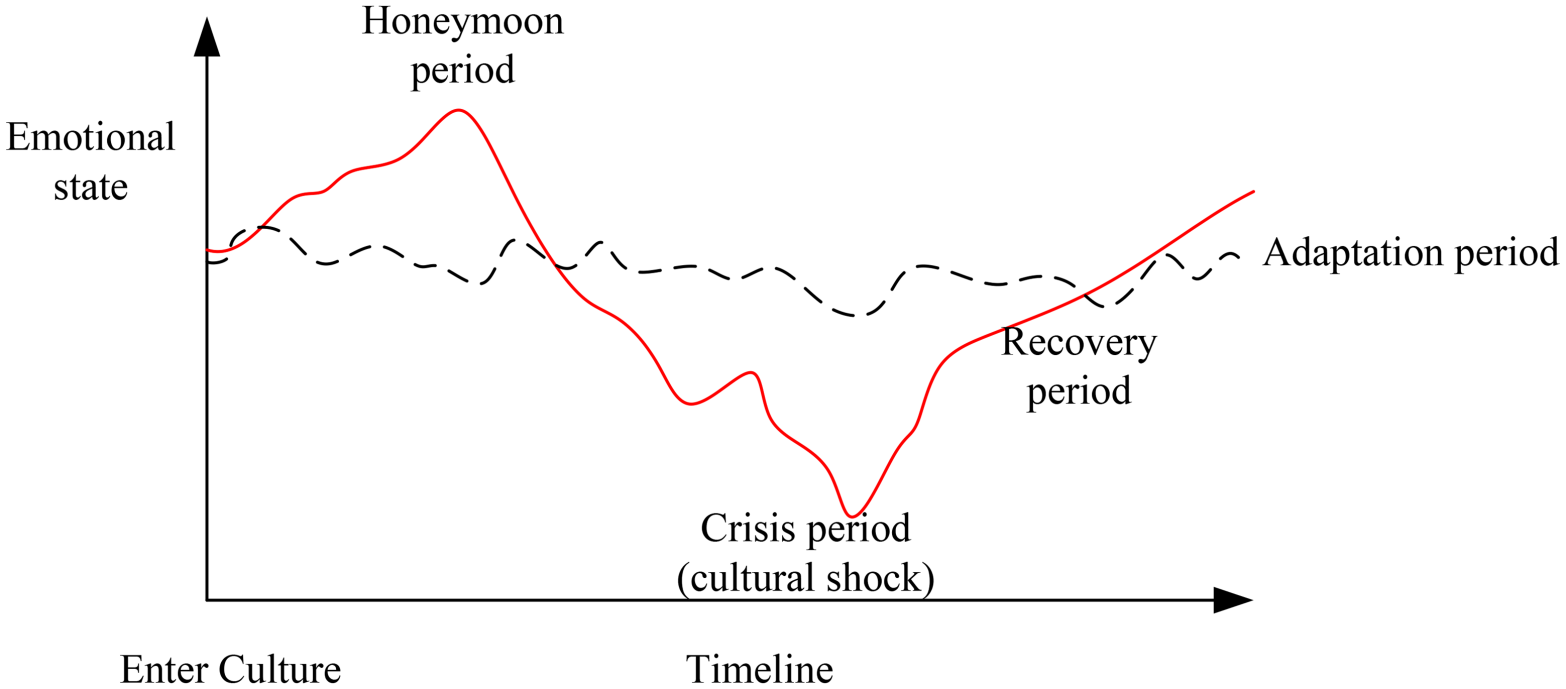
U-shaped pattern exhibited by the acculturation process.

The term cross-cultural refers to the interaction between groups with two or more different cultural backgrounds. Cross-cultural adaptation refers to the process by which participants adapt to a new environment that features a cultural background different from their own. Intercultural adaptation is a dynamic process that refers to the process of intentional selection and behavioral adjustment as participants move from their own culture to that of the current environment and gradually develop effective communication skills (Kashyrtsev, 2021). As early as 1960, a theory had been proposed that an individual would generally undergo a certain process when transitioning from a heterogeneous culture from a parent culture, which can be summarized as follows: there is an initial honeymoon period, following which conflict between the two cultures gradually produces incompatibility, leading to the crisis period; subsequently, the recovery period features the beginning of rational understanding and the expectation of changes, and the process concludes with the adaptation period (Wilhelm and Weber, 2018). In fact, to understand the process of cross-cultural adaptation, a U-shaped model may be more intuitive (as shown in Figure 1). The point of this model is that when an individual transitions to a heterogeneous culture from a parent culture, he or she experiences various discomforts resulting from the initial sense of novelty, faces the impact of cultural changes, and finally, in a slow and grinding process of adaptation, he or she develops an initial sense of comfort in the parent culture. Life changes associated with the parent culture are represented by dashed lines, while life changes associated with the heterogeneous culture are represented by wavy lines (Hyunchul, 2020).

According to Figure 1, people make psychological adjustments and learn to make use of the elements of the second culture when faced with a changing cultural situation, and this process constitutes cross-cultural adaptation. The psychological state of anxiety, loss, or fear that a person experiences when transition from a maternal environment to an unfamiliar environment is called culture shock (Maceratini, 2019).

Following a brief period of novelty, many challenging issues inevitably arise in the lives of students. These problems not only affect the success or failure of their studies but also their evaluations of the host country after returning home, thereby affecting China’s international image. International students in China usually face the following common problems in cross-cultural communication, study, and life (Carvalho, 2019).

The first such problem is the language barrier. Generally, international students who travel to China are at an early stage of learning Chinese, and it is not easy for them to communicate with Chinese people in Chinese. Apart from the teaching staff at the school, once students leave the school and come into contact with more people in society, they cannot easily communicate with a considerable number of people in English. This situation can lead to psychological frustration, i.e., the use of insincere words or repeated failures in communication (Gostev and Belous, 2019). In terms of language learning, foreign students who are familiar with their native languages may find it very difficult to develop an initial familiarity with square and four-color Chinese characters. This difficulty creates many problems and obstacles in the early stages of learning a language.

Second, the living environment is different. There are differences between China and foreign countries in terms of weather, diet, public environment, etc. These changes place pressure on people, and they all must be adapted to by international students in China (Hardecker, 2019).

**Figure 2:**
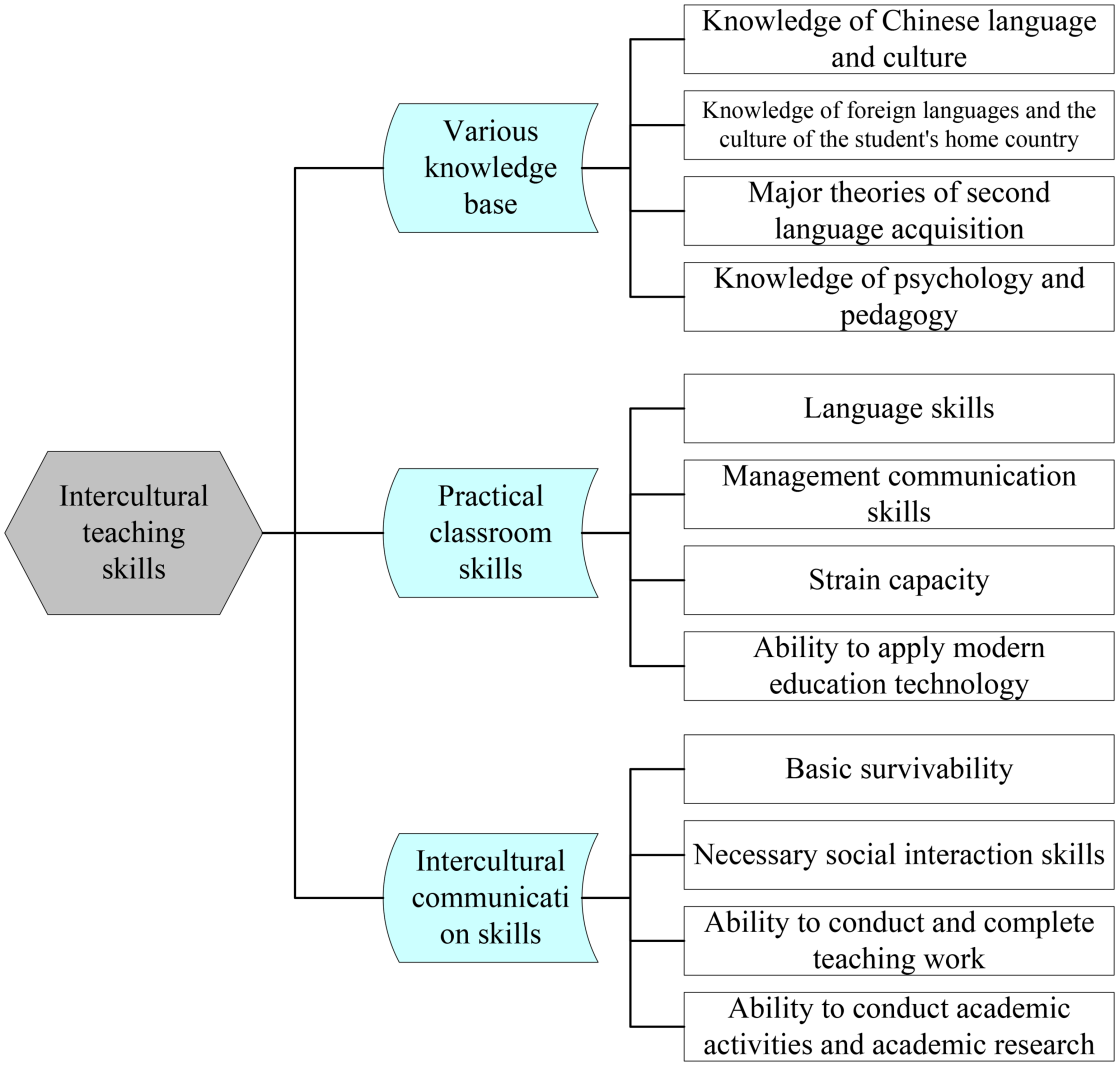
Composition of cross-cultural teaching competence in education.

Third, different cultural backgrounds are a factor. Many international students experience “culture shock” because they have left a familiar environment and transitioned to an unusual and markedly different cultural context. Differences in lifestyles, values, and beliefs between international students and Chinese culture can lead to culture shock. For example, Chinese people are quite reserved and introverted, while foreigners are more open and direct. Although most teachers who teach international students are trained to address both Chinese and Western cultures, the teaching process inevitably contains traces of Chinese-style teaching. China is a country that emphasizes collectivism, which conflicts in many ways with Western values of individual superiority. This situation can make it difficult for them to interact with other people. In the context of international education, China’s foreign education and teaching have entered a period of rapid development, and the problem of foreign teachers has attracted attention from the academic community. Teachers should have the ability to teach across cultures. This cross-cultural teaching ability is developed as shown in Figure 2.

**Figure 3:**
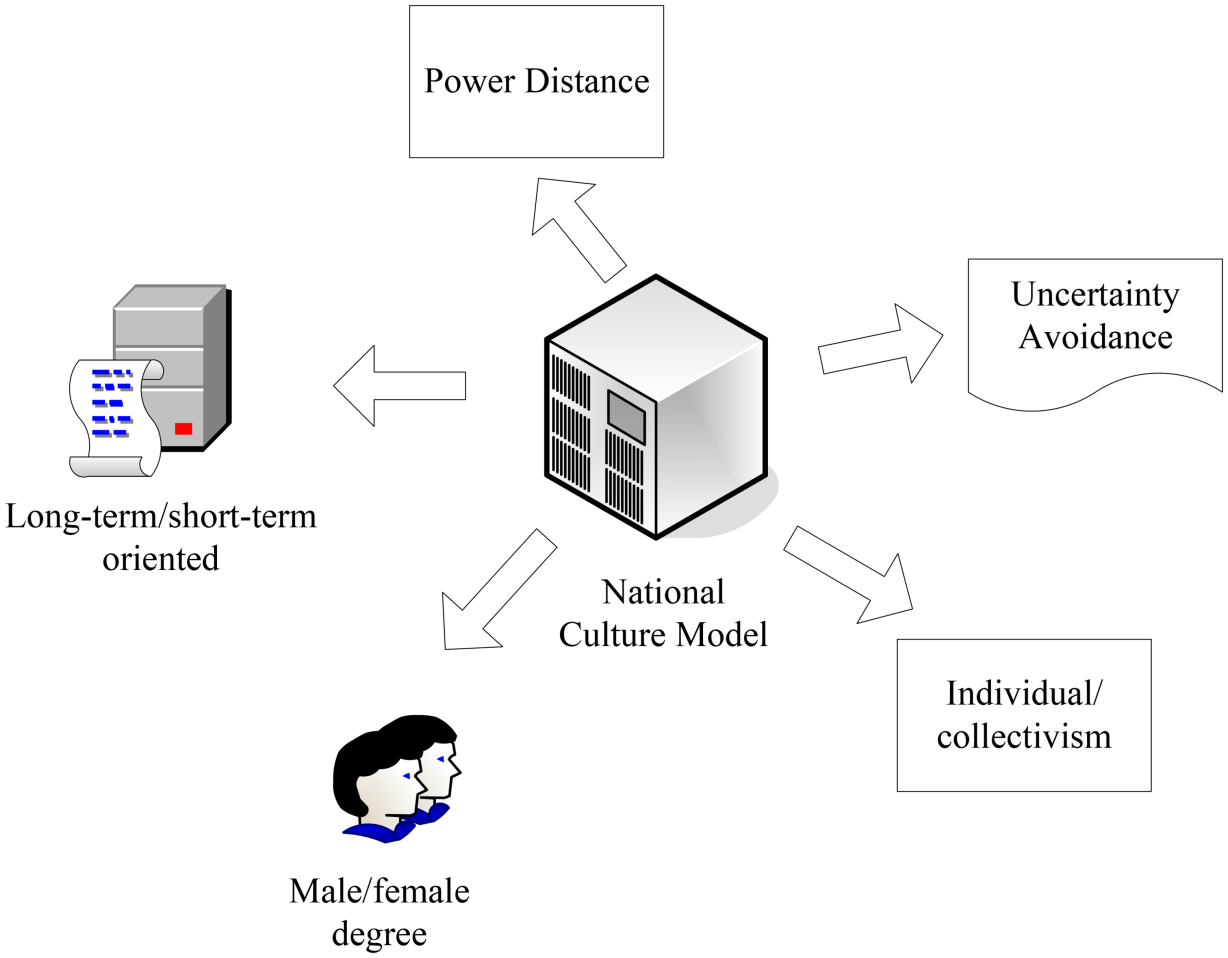
National culture model.

The cross-cultural management model—national cultural model, which mainly includes five dimensions, i.e., power distance, individualism and collectivism, masculinity and femininity, uncertainty avoidance, and long-term orientation and short-term orientation, as shown in Figure 3. Culture is not merely an individual characteristic but rather a mental program shared by people in the same environment.

### Pivot deconstruction

There are many cultural categories, and the factors affecting cross-cultural communication are also complex. A comprehensive understanding of the intercultural adaptation of international students from different countries and in-depth exploration of the factors that affect the intercultural adaptation of international students are beneficial with respect to enriching the theoretical research concerning the intercultural adaptation of international students. Association rules are expressions of the form A⇒B. Let S represent a set of items, and in a given transaction database, each nonempty subset of S represents a transaction (Transaction) t with a unique identifier IDT (Transaction ID) corresponding to it.

In real life, the information needed and the information used contain a large amount of data. This kind of data, which is not known in advance and has potential value, is exactly what people need, and association rules accomplish precisely this task (Wang et al., 2020; Li and Zhang, 2021) because they can uncover every hidden relationship. With respect to these seemingly unrelated items, there seem on the surface to be no relationships, but association rules can find subtle relationships among them. Association rules were proposed in 1993 and play an important role in data mining (Hassan et al., 2019; Hong et al., 2019a; Liao and Ho, 2021). Many algorithms have been proposed on this basis. Now, the definition of the association rule is given:

Definition 1: If.

(1) $$\text{S}=\left\{s_{1},s_{2},\ldots ,s_{\text{n}}\right\}$$

where S is the set of items, and the transaction set of the transaction database is B. Each transaction T is contained in the set of items S, i.e., T⊆S, and the itemset A is contained in the transaction T, i.e., A⊆T. Association rules are implications of the form A⇒B, in which context, A⊂S,B⊂S, and A∩B=∅.

Definition 2: The definition of support in the form of expression A⇒B is that both A and B are included in database D; that is, the proportion of the owned quantity in the total database D can be expressed as

(2) $$\mathrm{Suppor}t_{A\Rightarrow B}=p\left(A\cup B\right)$$

Additionally, for confidence, this proportion can be formally expressed as follows:

Definition 3: The confidence level in the form of Expression A⇒B is defined as the ratio of the number of database D that contains B to the total database D under the premise of containing A, which can be expressed as follows:

(3) $$\mathrm{Confidenc}e_{A\Rightarrow B}=p\left(B|A\right)$$

In the mining process of the algorithm, only rules that are greater than the minimum level of support and confidence are valuable to the decision-maker. It is necessary for the algorithm to mine these association rules more efficiently. Support can count the number of association rules included in the transaction database (Xue and Wang, 2016; Yang et al., 2021). The confidence is to mine a frequent itemset under the condition of support, which can be used to account for the importance and reliability of the rules. The frequent itemsets thus excavated may only satisfy the minimum level of support but not the minimum level of confidence, which is called the weak association rule. If both $\mathrm{Suppor}t_{A\Rightarrow B}$ and $\mathrm{Confidenc}e_{A\Rightarrow B}$ are satisfied, the rule can be called a strong association rule.

Association rule mining is one of the main techniques used in data mining. Because of its simple algorithm and wide range of applications and because the rules it excavates are easy for people to interpret and accept, this approach has received a great deal of attention. Association rule mining can discover interesting associations or correlations among itemsets that are embedded in a large amount of data. This approach focuses on solving the association rule mining problem when the target attribute category attribute is known (Zeng et al., 2019; Zhihan and Houbing, 2021). The mining of association rules is a two-step process. (1) Find all frequent itemsets; by definition, these itemsets appear at least as frequently as a predefined minimum support count. (2) Generate strong association rules from frequent itemsets; by definition, these rules must satisfy minimum levels of support and confidence.

Association rule-based classification is a technique that uses a set of association rules to classify transactions. Using the classification association rule, mining algorithm can allow the training of the classifier to be completed more quickly than the classification rule mining algorithm and can control it very effectively when training with a high-dimensional training set. Each classification rule can be expressed in the following form:


(4) $$R:A\to F_{i}$$

The left side of the rule is called the rule antecedent, which is a combination of feature tests. The right side of the rule is called the rule consequent and contains the label for the predicted class $F_{i}$. A rule R can be said to cover T if its priors correspond to properties of transaction T. Given a transaction dataset D and a classification rule A→f, the support of the rule is defined as the percentage of firms in T that contain a set of itemsets $\left(A\cap f\right)$, and confidence is defined as the percentage of transactions in T that contain a set of itemsets $\left(A\cap f\right)$. Formally, these two metrics are defined as follows:


(5) $$\mathrm{Support}\left(r\right)=\frac{\left|A\right|}{\left|D\right|}$$

(6) $$\mathrm{Confidence}\left(r\right)=\frac{\left|A\cap c\right|}{\left|D\right|}$$

where |A| is the number of records that satisfy the antecedents of the rule, |A∩f| is the number of records that satisfy both the antecedent and the consequent of the rule, and D is the total number of records.

The basic idea of PCA is to reduce the dimensionality of the high-dimensional variable space while guided by the principle of ensuring the least loss of data information. Through certain transformations, multiple correlated variables are transformed into a few uncorrelated variables to simplify the analysis.

Suppose that A is a q × p-dimensional data matrix, where each column corresponds to a variable and each row corresponds to a sample. The matrix A can be decomposed into the sum of the outer products of p vectors, namely:


(7) $$A=t_{1}m_{1}^{U}+t_{2}m_{2}^{U}+\ldots +t_{p}m_{p}^{U}$$

where $t_{i}$ is called the score vector and $m_{i}$ is called the load vector. The score vector of A is also called the pivot of A.

Each score vector is orthogonal, that is, for any *i* and *j*, when i≠j, this situation satisfies:


(8) $$t_{i}^{U}t_{j}=0$$

The load vectors are also orthogonal, and the length of each load vector is 1; that is, the following two formulas are satisfied:


(9) $$m_{i}^{U}m_{j}=0\;i\neq j$$

(10) $$m_{i}^{U}m_{j}=1\;i=j$$

Multiplying both sides of Formula (7) to the right by $m_{i}$ simultaneously, the following formula can be obtained:


(11) $$Am_{i}=t_{1}m_{1}^{U}m_{i}+t_{2}m_{2}^{U}m_{i}+\ldots +t_{i}m_{i}^{U}m_{i}+\ldots +t_{m}m_{m}^{U}m_{i}$$

Substituting Formulas (9) and (10) into Formula (11), the following can be obtained:


(12) $$t_{i}=Am_{i}$$

Formula (12) shows that each estimated vector is a projection of data matrix A in the direction of the load vector corresponding to that estimated vector. The vector length $t_{i}$ reflects the coverage of data matrix A in the $m_{i}$ direction. The longer the length is, the greater the coverage or deviation of A in the $m_{i}$ direction. If the score vector is sorted in descending order of length as shown below,


(13) $$t_{1}\geq t_{2}\geq \ldots \geq t_{p}\text{,}$$

The load vector $m_{1}$ represents the direction in which data matrix A changes the most. $m_{2}$ is perpendicular to $m_{1}$, representing the second largest direction of change in data matrix A, and $m_{p}$ represents the direction in which data matrix A changes the least.

If there is some degree of linear correlation among the variables in data matrix A, then the changes in A are mostly in the direction of the first few load vectors. The projection of data matrix A onto the last few load vectors is small, which is likely mainly due to measurement noise. This approach allows people to write the pivoted data matrix A in the form of the following formula:


(14) $$A=t_{1}m_{1}^{U}+t_{2}m_{2}^{U}+\ldots +t_{k}m_{k}^{U}+W$$

where W is the error matrix, representing a change in A in other load vector directions. In many practical applications, *k* is often much smaller than p. If the error matrix W is ignored, this situation can be approximately expressed as follows:


(15) $$A=t_{1}m_{1}^{U}+t_{2}m_{2}^{U}+\ldots +t_{k}m_{k}^{U}$$

Principal component analysis can be computed using the nonlinear iterative least squares algorithm (NIPALS), which computes each pivot element of the data matrix A in turn. First, calculate the first score vector $t_{1}$ and the first load vector $m_{1}$. Next, subtract their outer product from the data matrix to obtain the error matrix $W_{1}$. Then, calculate the second score vector $t_{2}$ and the second load vector $m_{2}$ from the error matrix:


(16) $$W_{1}=A-t_{1}m_{1}^{U};\;W_{2}=W_{1}-t_{2}m_{2}^{U};\ldots \ldots$$

This calculation continues until all pivots in the data matrix have been calculated.

From a statistical perspective, to determine whether the data contain faulty information regarding the process, a hypothesis test can be conducted by creating a statistic to determine whether the process data deviate from the master meta-model.

The essence of communication is the exchange of information, listening to each other’s opinions, and interpersonal communication regarding work and feelings. Under the ongoing global trend of the internationalization of higher education, the internationalization of student sources is becoming increasingly obvious (Hong et al., 2019b). The education of international students is no longer merely a decoration of the “internationalization” of a university but one of the most important symbols of the overall development of the university. The education of international students is not only part of a university’s “soft power” but also one of the most basic and important factors involved in the process of university internationalization. Developing the education of students studying in China, on the one hand, can create a university environment that facilitates cross-cultural exchange and create an international atmosphere for cultivating global professionals. On the other hand, it can promote the internationalization of university activities and improve university management. In the context of the ongoing process of internationalization in higher education, the school must revise its concepts and management system and combine the development of international student education with the general promotion of internationalization and the internal construction of the school. Without bypassing the contradictions and difficulties of reality, it must deepen the educational reform, make bold innovations of the system, and actively explore new ways and methods of teaching and management with respect to international students.

## Communication action theoretical model construction and questionnaire design

### Model construction

**Figure 4:**
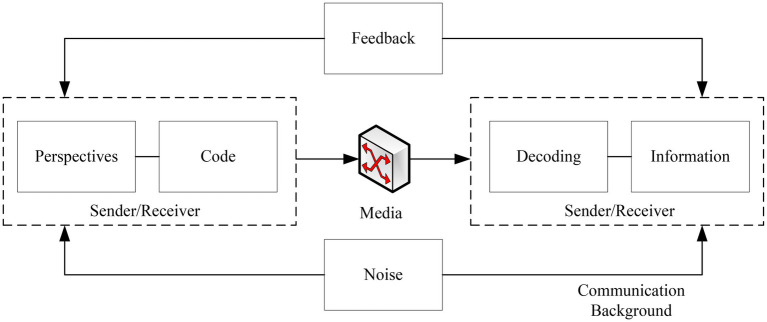
Communication element model construction.

In the actual implementation of the communication action model, there is a mutual expectation. That is, the subject makes efforts to engage in the action and proposes requirements for the effectiveness of the speech act, and the subject also expects the other party to make efforts to engage in the action and propose requirements for the effectiveness of the speech action. Once this validity claim is challenged, a process of rational debate is necessary. Each party supports its own validity claims with better arguments, counters the other’s validity claims, and strives to continue the communication action. This situation represents a form of rationality that is different from deliberative rationality, including the ability to reflect, criticize, and argue. It is a dialog-based rationality and a communicative rationality, as shown in Figure 4.

**Figure 5:**
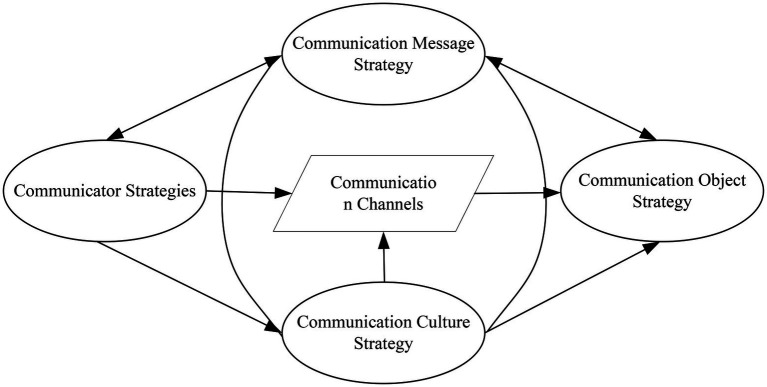
Five strategies for effective communication.

The essence of communication is the transmission of information. The degree of reliability and accuracy demonstrated during this process is called effective communication. In brief, after the signal is received *via* the channel, whether or not the signal is distorted, of course, noise and distortion are inevitable (Li et al., 2015). If this process has an impact on effective communication, the influencing factors are multifaceted, both including microscopic factors, such as noise and media, and macroscopic factors (Li et al., 2018). This process depends not only on the coding ability exhibited by the subject when conveying information but also on the decoding ability exhibited by the object receiving the information. Five strategies for effective communication are shown in Figure 5.

### Deconstruct methods

#### Literature research

This paper collects literature concerning intercultural adaptation and communication, organizes and summarizes relevant theories concerning intercultural adaptation and communication, and summarizes the general difficulties and problems encountered by students in intercultural adaptation communication in the context of international education (Ilkevich et al., 2021; Li et al., 2021).

#### Questionnaire method

This study takes international students associated with the international department of a middle school in a city as its research object. According to the questionnaires issued, by collecting the communication and learning status of 120 international students, international students’ adaptation to their living environment, education and teaching environment, psychological characteristics, and interpersonal communication were analyzed. These students come from many different countries and regions and have rich and diverse cultural backgrounds, and the results are thus representative to some extent. Information concerning the international students who participated in this survey is shown in Tables 1, 2.

**Table 1:** Basic information concerning the survey subjects.

Projects		Number of people	Frequency (%)
Gender	Male	60	50
	Female	60	50
Age	20–25	70	58.3
	26–35	29	24.2
	≥36	21	17.5

**Table 2:** Personal experiences of respondents.

Projects		Number of people	Frequency (%)
Professional background	Chinese as a Foreign Language	90	75
	Other	30	25
Exchange time	1–6 months	10	8.3
	6–12 months	55	24.2
	12–24 months	21	17.5
	≥1 year	34	28.3

In terms of gender, according to the survey, among the 120 international students, 50% (60) were male and 50% (60) were female. Regarding the second item concerning age, the group in their twenties accounted for a large proportion of the total, i.e., 58.3% (70 people). A total of 75% (90 people) of the survey respondents studied Chinese as a foreign language during this period. Twenty-five percent (30 people) did not major in Chinese as a foreign language. Students who were not majoring in Chinese as a foreign language majored in English, education, linguistics, law, tourism management, etc.

## Situation and countermeasures of cross-cultural adaptation of international education students

### Adaptation of international students to the living environment on campus

**Figure 6:**
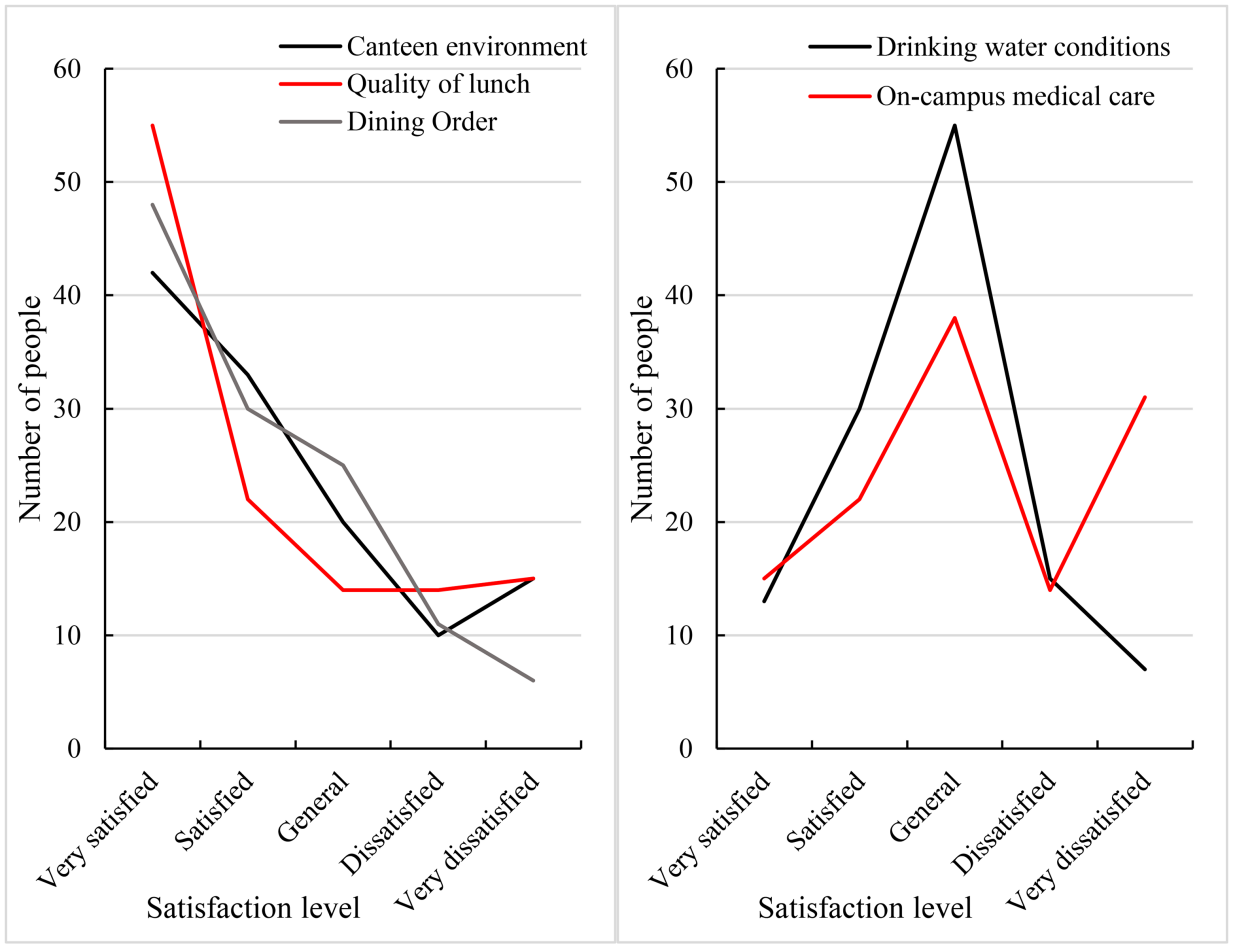
Survey results concerning overseas students’ satisfaction with dietary conditions, drinking water conditions, and on-campus medical conditions.

The cultural backgrounds of participants were rich and diverse, and the results are thus representative. Due to the relatively simple living environment on campus, this survey covered only lunch, drinking water, and on-campus medical care. This paper used the principles and methods of path analysis to reflect students’ adaptability *via* a survey of students’ satisfaction. In this context, students were very satisfied, indicating that their adaptability was the highest, as shown in Figure 6.

As seen in Figure 6, among the three indicators of canteen environment, lunch quality, and dining order, a larger proportion of people were relatively satisfied with their dietary conditions. Among participants, 45.8% (55 people) were very satisfied with the quality of lunch, and 24.1% (29 people) were very dissatisfied with the quality of lunch. Most of the students were also generally satisfied with the drinking water available in the school, 35.8% of whom were satisfied or very satisfied with the drinking water conditions, and 18.3% were dissatisfied and very dissatisfied. The degree of adaptability of students to on-campus medical care was not positive. However, the majority of students were also moderately satisfied with on-campus medical conditions. However, 37.5% of the students were dissatisfied or very dissatisfied with the on-campus medical care, and only 30.8% of the students were satisfied or very satisfied.

### Adaptation of international students to the educational and teaching environment

**Figure 7:**
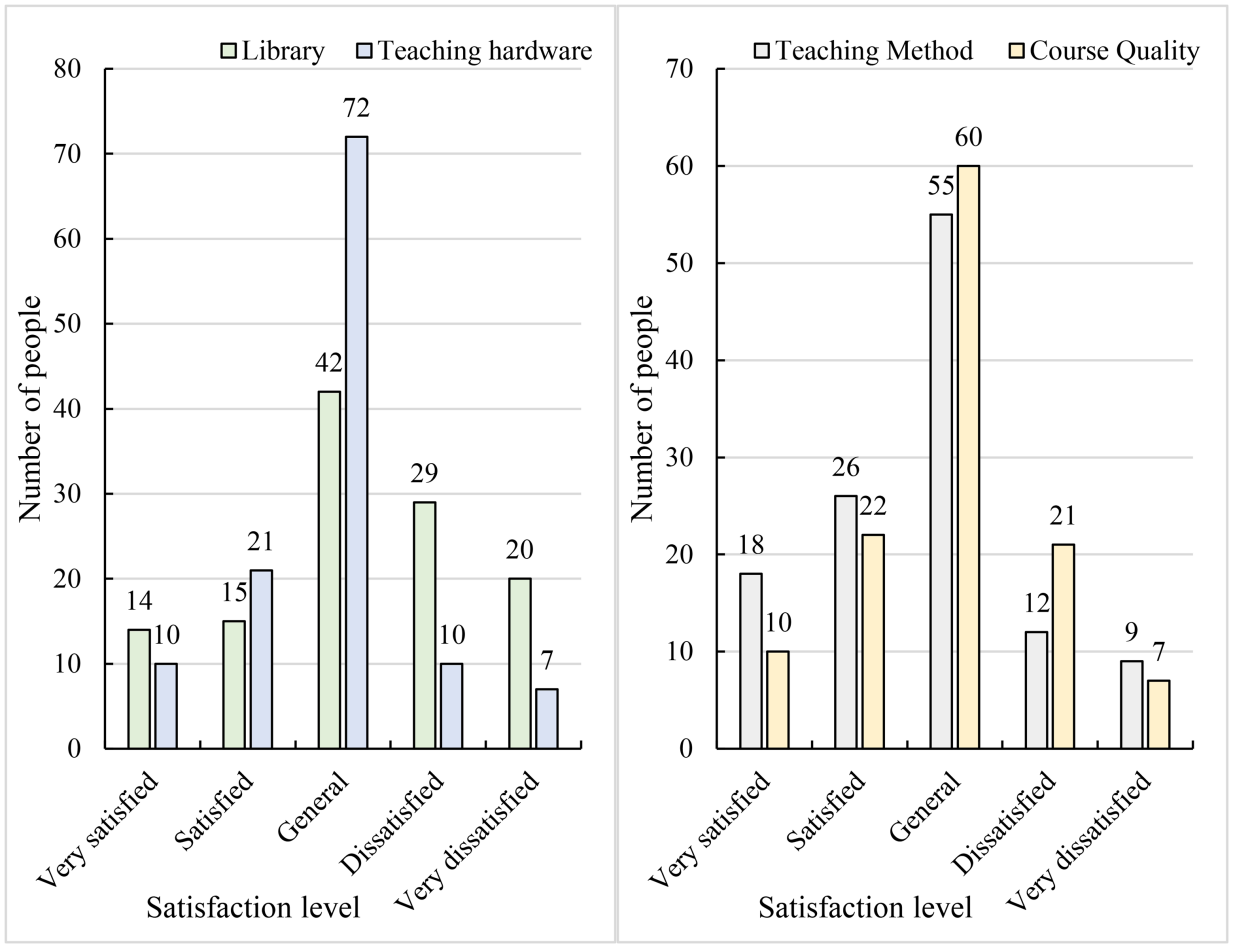
Satisfaction survey of overseas students concerning teaching facilities and teaching quality.

Figure 7 shows the results of the questionnaire concerning the satisfaction of overseas students with regard to teaching facilities and teaching quality.

Figure 7 shows that the school’s teaching hardware and facilities were relatively good. In terms of teaching methods, 36.7% of the students were satisfied or very satisfied, 45.8% of the students thought that the teaching methods were average, and 17.5% of the students were dissatisfied or very dissatisfied. In terms of course quality, 23.3% of the students noted that they were dissatisfied or very dissatisfied. This finding shows that students were not accustomed to this aspect of education and teaching. The results showed that although language barrier was one of the reasons for incomprehension, it was not the main reason, as shown in Table 3. Excessively deep class content was the main reason that students did not understand.

**Table 3:** Distribution of Chinese proficiency among international students in China.

Proficiency in Chinese	Frequency	Percentage (%)	Effective percentage (%)
Skilled	65	54.2	60.2
Unskilled	43	28.3	39.8
Missing	12	17.5	–

Students’ degree of adaptability to the teaching methods was relatively high. Most foreign students exhibited adaptability to the teaching methods, curriculum, class content, and teaching materials, likely because many of them came from Chinese cultural backgrounds. However, some students were uncomfortable or very uncomfortable with the teaching methods. Discomfort with teaching methods is often characterized by a very introverted, less talkative, and rebellious attitude, which is essentially a clash between Chinese and Western cultures.

### Psychological characteristics of international students and their adaptation to interpersonal communication

**Figure 8:**
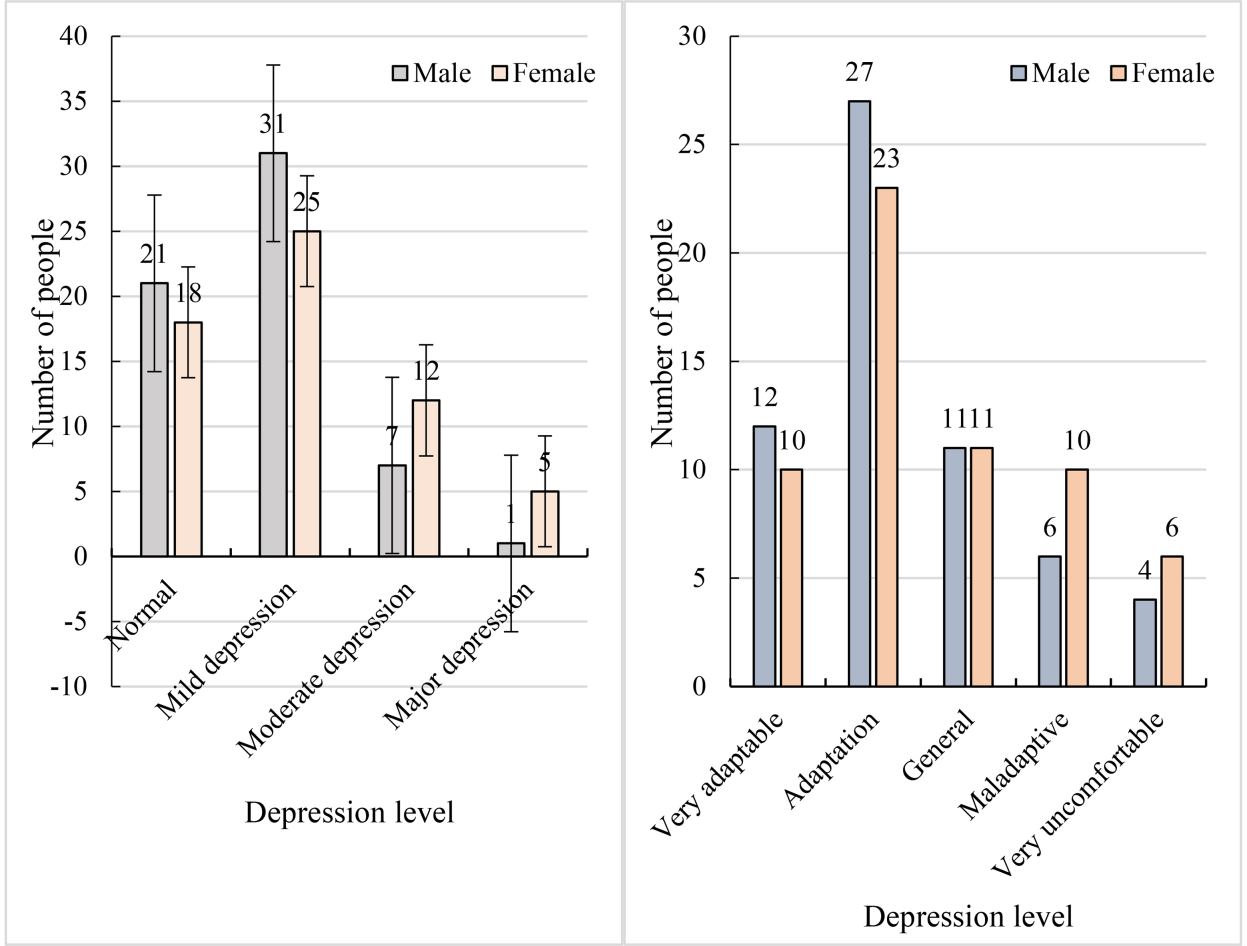
Psychological characteristics of international students and their adaptation to interpersonal communication.

Psychological adaptation is based on the individual’s emotional response and refers to the psychological health and life satisfaction of the subject of cross-cultural adaptation in the interaction. In cross-cultural interaction, when the adaptive subject has fewer or no negative emotions, such as depression, anxiety, loneliness, and depression, psychological adaptation can be said to have been achieved. Psychological adaptation is one of the important reflections of the state of cross-cultural adaptation. The psychological adaptation of international students refers to the process by which international students evaluate their sojourn experiences and the strategies they use to relieve stress in response to the stimulation of life changes in the cross-cultural transition. The results of adaptation vary in accordance with individual and situational factors and are reflected in the emotional and cognitive states of international students. In accordance with the psychological characteristics of ordinary students, this paper designs a psychological adaptation survey to judge the levels of psychological depression exhibited by international students. The degree of psychological adaptation is reflected in terms of depression levels. Degrees of depression can be divided into normal, mild depression, moderate depression, and severe depression. A summary of the survey results is shown in Figure 8.

The survey indicated that the psychological adaptability of girls is slightly worse than that of boys and that the depression levels exhibited by girls are slightly higher than those exhibited by boys. The survey showed that among boys, the numbers who exhibited normal psychology, mild depression, moderate depression, and severe depression were 21, 31, 7, and 1, respectively. Among girls, the numbers who exhibited normal psychology, mild depression, moderate depression, and severe depression were 18, 25, 12, and 5, respectively. According to a survey concerning the interpersonal communication of 120 international students in China, most international students (60%) were able to adapt to interpersonal relationships.

The analysis of the experimental results indicated that international students can solve the problems associated with cross-cultural adaptation in the following ways. (1) They can actively participate in interpersonal communication. On the one hand, schools and educational institutions must provide additional opportunities for foreign students to learn and communicate in new cultural environments. On the other hand, schools and educational institutions can train foreign students in cross-cultural communication skills, encourage them to cooperate and communicate, and enable them to appreciate the importance of communication. (2) When international students recognize cultural differences in their new environment, a better understanding of the new culture plays an important role in promoting their cross-cultural adaptation. (3) Students’ interest in studying in China can be increased. Their motivation to adapt to their new cultural environment is not high, and their interest is likely to be confined to one’s own home culture in a narrow way. To improve students’ interest in studying abroad, schools and educational institutions can learn more about the customs and culture of the destination country, such as food, festivals, and clothing. In addition, more opportunities can be provided to allow international students to participate in social activities and engage in more exchanges with Chinese people to enhance their interests further. To solve the problems associated with cross-cultural adaptation, we must also pay attention to the cultivation of cross-cultural communication awareness and the improvement of cross-cultural communication ability.

### Data testing

Structural equation modeling (SEM) is a type of empirical analysis used to investigate complex multivariate research data. SEM can be used to measure explicit variables to infer implicit variables, and the data can thus be used to test the validity of the hypothesis model. SEM has great advantages in measuring multiple variables.

A SEM hypothesis model was developed based on the identified indicators of international students’ intercultural adjustment in international education. According to the hypothesis model, three adaptation factors, namely, on-campus living environment, educational and teaching environment, and interpersonal interaction, are exogenous latent variables.

#### Reliability analysis

Before the sample data can be used for model fitting, the data must be tested for reliability and validity. The retest reliability method, the replicate reliability method, the half confidence method, and Cronbach’s α test were used to weigh the advantages and disadvantages of each method. SPSS 19.0 software was used to conduct Cronbach’s α test on the data obtained from the questionnaire survey. Table 4 shows that the Cronbach’s α coefficients of the three latent variables were > 0.8 and that the Cronbach’s α coefficients of the total table were also > 0.8, indicating that the data obtained *via* this questionnaire survey exhibited high reliability and could be used for the structural equation goodness-of-fit test.

**Table 4:** Reliability analysis.

Latent variables	Cronbach’s α
On-campus living environment	0.835
Educational and teaching environment	0.824
Interpersonal interaction	0.813

#### Validity analysis

Validity refers to the validity of the questionnaire results, and validity testing is used to test the objectivity of the questionnaire results. There are various types of validity, which can be divided into content validity, practical validity, and structural validity; these three types of validity analysis have different perspectives and focuses. This paper mainly analyzed structural validity, and the results showed that the structural validity of the questionnaire data is good. The KMO test was conducted for three variables: school life environment, educational and teaching environment, and interpersonal interaction. The inspection results are shown in Table 5.

**Table 5:** KMO measures.

Test methods	Statistics	Value
KMO measure	KMO	0.836
	Approximate Chi-square	2.222

## Conclusion

When international students enter the cultural environment of mainland China with an understanding of their own country and regional culture, the impact of cultural differences can lead to problems of adaptation. Habermas’s communication action theory aims to establish a universal “normative foundation” or standard to describe, analyze, critique, and evaluate the structure of modern society. Cultural and conceptual differences exist between groups of different languages and ethnicities. These differences are reflected in all aspects and create problems for educational administration. Quantitative research shows that language barriers and cultural differences are the main difficulties that international students face in the context of cross-cultural adaptation. International students in China can encounter more or fewer problems and difficulties throughout the process of cross-cultural adaptation. Although many situations are common among such students, they vary from person to person and from place to place, which is largely the result of each person’s own psychological quality. Therefore, the feelings and experiences of such individuals exhibit a great deal of variety. The survey revealed that the psychological adaptability of girls is slightly less than that of boys and that the levels of depression exhibited by girls are slightly higher than those exhibited by boys. To promote the internationalization of education, it is not sufficient merely to recruit international students *per se*; more importantly, the school should strengthen the top-level design of internationalization as a whole to incorporate the concept of internationalization fully into the school’s teaching, scientific research, teacher training, discipline construction, logistics services, student management, and other aspects.

## Data availability statement

The original contributions presented in the study are included in the article/supplementary material; further inquiries can be directed to the corresponding author.

## Author contributions

YL: writing. YS: editing. and YY: translating. All authors contributed to the article and approved the submitted version.

## Funding

This work is supported by the research center of the humanities and Social Sciences in Sichuan Province—the international communication research center of Sichuan wine culture “the current situation and path of Chinese Baijiu culture’s external communication (CJCB2020-11), and Key research base of philosophy, and the key research base of philosophy and Social Sciences in Sichuan Province—the key project of Sichuan Primary and secondary school teacher professional development research center “Research on the training path of rural excellent teachers under the background of educational modernization (PDTR2020-01), and the special scientific research project of on-the-job doctoral students of Chengdu Normal University in 2020” Research on the Framework of Teaching Knowledge System under the Background of Artificial Intelligence” (ZZBS2020-10).

## Conflict of interest

The authors declare that the research was conducted in the absence of any commercial or financial relationships that could be construed as a potential conflict of interest.

## Publisher’s note

All claims expressed in this article are solely those of the authors and do not necessarily represent those of their affiliated organizations, or those of the publisher, the editors and the reviewers. Any product that may be evaluated in this article, or claim that may be made by its manufacturer, is not guaranteed or endorsed by the publisher.

## Appendix

Appendix I

Questionnaire on intercultural adaptation of International Education Students.

1. Are you satisfied with the canteen environment on campus?

A. Very satisfied, B. Satisfied, C. Average, D. Dissatisfied, F. Very dissatisfied.

2. Are you satisfied with the quality of lunch on campus?

A. Very satisfied, B. Satisfied, C. Average, D. Dissatisfied, F. Very dissatisfied.

3. Are you satisfied with the dining order on campus?

A. Very satisfied, B. Satisfied, C. Average, D. Dissatisfied, F. Very dissatisfied.

4. Are you satisfied with the drinking water conditions on campus?

A. Very satisfied, B. Satisfied, C. Average, D. Dissatisfied, F. Very dissatisfied.

5. Are you satisfied with the medical conditions in the school?

A. Very satisfied, B. Satisfied, C. Average, D. Dissatisfied, F. Very dissatisfied.

6. Are you satisfied with the teaching facilities?

A. Very satisfied, B. Satisfied, C. Average, D. Dissatisfied, F. Very dissatisfied.

7. Are you satisfied with the teaching quality?

A. Very satisfied, B. Satisfied, C. Average, D. Dissatisfied, F. Very dissatisfied.

8. Do you like to discuss and exchange problems with your classmates in class?

A. Like, B. Do not like.

9. Is the classroom atmosphere relaxed and pleasant?

A. Yes, B. No.

Appendix II

Investigation on psychological adaptation of international education students (2).

1. Your gender is:

A. Male, B. female.

2. Your current grade is:

A. Senior 1, B. Senior 2, C. Senior 3.

3. Frequency of late arrival, absenteeism, and early leave during school since enrollment.

A. No or very few (less than 3 times per semester), B. 1–2 times per week on average. C. 3–4 times a week on average, D. This happens more than half the time every week.

4. If you are often not interested in a course or want to be absent, the main reasons are:

A. Not interested in the course, B. Not interested in the teachers, C. Lack of motivation, D. Influenced by surrounding students, E. others_________________.

5. Do you feel academic pressure during the semester?

A. Very stressful, B. A little stressful, C. No pressure.

6. Do you feel anxious when communicating with your classmates?

A. Yes, B. No.

7. Who do you usually talk to when you encounter difficulties?

A. Parents, B. Classmates, C. Friends, D. Teachers, E. Self-relief.
